# The Balance of Ketoacids α-Ketoglutarate and α-Ketoglutaramate Reflects the Degree of the Development of Hepatoencephalopathy in Rats

**DOI:** 10.3390/ijms252413568

**Published:** 2024-12-18

**Authors:** Yevgeniya I. Shurubor, Anton A. Keskinov, Vladimir S. Yudin, Boris F. Krasnikov

**Affiliations:** 1 Centre for Strategic Planning of FMBA of the Russian Federation, Pogodinskaya St., Bld. 10, 119121 Moscow, Russia; keskinov@cspfmba.ru (A.A.K.); vyudin@cspfmba.ru (V.S.Y.); 2 Department of Biochemistry and Molecular Biology, Faculty of Medicine, N.I. Pirogov Russian National Research Medical University, 1 Ostrovitianova Str., 117997 Moscow, Russia

**Keywords:** biomarker, hepatoencephalopathy, α-ketoglutarate, α-ketoglutaramate

## Abstract

Hepatoencephalopathy (HE) is a liver disease that can lead to brain pathology and the impairment of human cognitive abilities. The objective assessment of HE disease severity is difficult due to the lack of reliable diagnostic markers. This paper examines the background to the emergence of HE markers and provides a brief overview of research results indicating the diagnostic value of potential markers isolated from a wide range of metabolites analyzed. It has been suggested that metabolites of the glutamate–glutamine (Glu-Gln) cycle, α-ketoglutarate (αKG), and α-ketoglutaramate (αKGM) can act as such markers of HE. The informative value of these markers was revealed during a comparative analysis of the distribution of αKG and αKGM in samples of the blood plasma and tissues (liver, kidneys, and brain) of rats exposed to the strong hepatotoxin thioacetamide (TAA). A comparative analysis of the balance of αKG and αKGM, as well as their ratio (αKG/αKGM) in the examined samples of blood plasma and animal tissues in these models, revealed their diagnostic value for assessing the severity of HE and/or monitoring the recovery process.

## 1. Introduction

Hepatoencephalopathy (HE) is a neurocognitive pathology that develops as a result of toxic brain damage caused by the metabolic dysfunction of the liver, and is expressed in the form of a wide range of neurological and psychiatric disorders, including changes in memory, intellectual, behavioral, emotional manifestations, and psychomotor skills [1,2]. The nature of the development of HE can be chronic or acute. In severe forms of HE, the fatal outcome can reach ~75% [3]. The importance of drawing attention to this problem is due to the massive nature and annual increase in the number of patients with HE in the world, including in economically developed countries. For example, in the USA for the period from 2005 to 2009 (data for a more current period of time are not available in the public literature), the annual number of hospitalizations of patients with HE was 110,000, and the cost of their hospital treatment in clinics annually amounted to up to USD 1 billion [4].

The pathophysiology of HE is complex and is often accompanied by hyperammonemia, cerebral dysfunction, and inflammation. Although the exact mechanisms of HE development are still not entirely clear, many researchers believe that the root cause is a stable and long-term increase in blood ammonia levels [5,6,7]. Under physiological conditions, ammonia in the liver is converted into urea and excreted from the body. However, in liver failure, it can accumulate in the blood, penetrate the blood–brain barrier, and affect the level of glutamine (Gln), the excess of which leads to the swelling of astrocytes, intracranial hypertension, and cerebral edema [5,6]. Increased blood ammonia levels are due in part to the activity of the enzyme glutamine synthetase and the glutamate–glutamine (Glu-Gln) cycle. In addition to ammonia and Gln, elevated levels of glutamate (Glu) have also been implicated in the development of various forms of HE. As Glu concentration increases and its absorption decreases, this amino acid becomes toxic and triggers a mechanism leading to cerebral edema [7]. The development of HE can also be promoted by oxidative stress, neurosteroids, systemic inflammation, increased levels of bile acids, and impaired lactate metabolism [5,6]. In addition, the development of HE is accompanied by changes in the levels of neurotransmitters, coenzymes, amino acids, metabolites of the tricarboxylic acid (TCA) cycle and associated metabolites, including α-ketoglutaramate (αKGM) and α-ketoglutarate (αKG) [8,9,10,11,12].

The diagnosis of HE is often difficult due to the large number of overlaps in the clinical picture with other diseases of a metabolic, neurological, or mental nature. Unfortunately, there are not yet markers that simultaneously have good sensitivity, correlate with the severity of the disease, and also have a high prognostic value and can adequately monitor the body’s response to therapeutic intervention in HE medical practice.

This review discusses the most common approaches in diagnosing HE, suggests the role of αKG and αKGM as potential biomarkers for the diagnosis of hyperammonemia and HE, and discusses options for laboratory synthesis of commercially unavailable αKGM and optimal methods for analyzing αKG and αKGM in biological samples. A comparative analysis of previous clinical studies of the biological samples of tissues and fluids obtained from patients, as well as preclinical studies on animal model systems, suggests the possibility of using the balance of αKG and αKGM, namely, the αKG/αKGM ratio, as a diagnostic marker of the severity and/or state of remission of HE.

## 2. Approaches to Diagnosing HE

When diagnosing HE, data from clinical tests and/or psychophysiological characteristics of patients are usually used [13]. At the same time, the results of the clinical diagnosis of HE are often controversial, since the metabolic picture of the development of pathology for each patient is very individual. For a more accurate diagnosis of HE, in addition to the results of clinical tests, it is desirable to have additional information on the extent of liver and brain damage based on neuropsychological and/or neurophysiological studies, as well as an assessment of the patient’s cognitive characteristics.

Thus, laboratory tests usually include a complete blood count, measurement of electrolytes, glucose levels, and assessment of the functional activity of the liver and kidneys. However, due to the complex picture of HE, these data do not always allow for a reliable diagnosis of the disease. Elevated levels of ammonia in the blood also do not always correlate with the severity of the disease, so it is rarely used for diagnostic purposes [14]. In addition to ammonia, the levels of a number of metabolites may be increased or altered in the blood of patients with HE, including αKG, αKGM, Gln, Glu, cyclic guanosine monophosphate (cGMP), interleukins-6 and -18 (IL-6, IL-18), and 3-nitrotyrosine, which has previously been positioned as a possible marker for the diagnosis of HE [10,12,15,16].

With the development of HE, patients experience a wide range of nonspecific neurological and psychiatric manifestations [1,7]. At the initial stages of the disease, changes are noted when patients undergo psychometric tests: decreased attention, working memory, speed of psychomotor movements, visuospatial abilities, electrophysiological parameters of the brain, etc. Neuropsychometric tests are used to identify disorders associated with decreased attention, visuospatial abilities, fine motor skills, and memory. However, the listed brain dysfunctions are also similar to other cognitive disorders, so these tests are difficult to classify as specific [17].

When conducting neurophysiological tests, the physiological functions of the brain and the hyperactivity of the basal ganglia and white matter are assessed, and the dynamics of brain swelling are monitored. As a rule, methods such as electroencephalography, cerebral magnetic resonance, cerebral spectroscopy, etc., are used [18,19]. However, the listed tests are not specific for identifying disorders associated with liver dysfunction, so there remains a need for additional tests. Such auxiliary methods for diagnosing HE include magnetic resonance imaging, which can detect cerebral edema, as well as a number of other anomalies caused by the development of HE or other pathologies. The disadvantage of this method is its weak correlation with the severity of the disease [20]. To identify the mild stage of HE development, electroencephalography is sometimes used, which is also not entirely specific, since the results obtained may be influenced by other metabolic disorders [17].

Thus, the prompt diagnosis of HE requires potential markers, the criteria for which are specificity, speed, and ease of diagnosis.

## 3. Relationship Between Hyperammonemia and HE and the Functioning of the Glu-Gln Cycle

**Figure 1:**
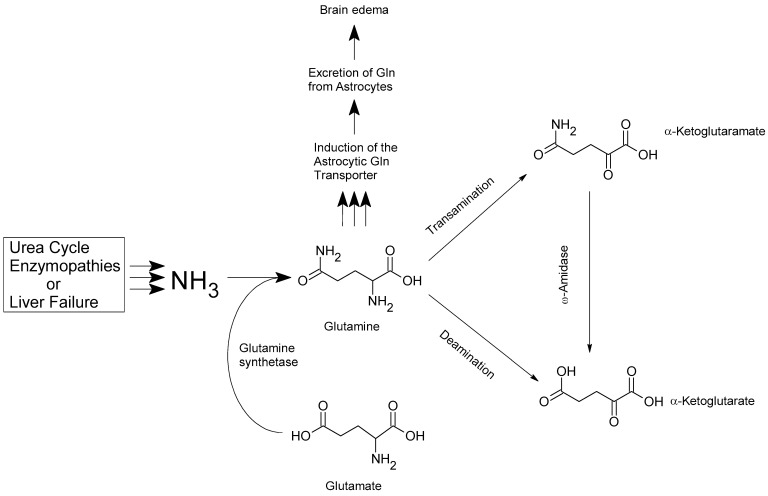
Alternative pathways affecting Gln homeostasis in the hyperammonemic brain.

The release of excess ammonia due to urea cycle enzymopathies or liver failure leads to increased levels of Gln, the content of which, in the brain, correlates with the severity of neurological symptoms. Alternative pathways affecting Gln homeostasis in the hyperammonemic brain include the deamination and transamination of Gln to form αKG and αKGM, as well as changes in the high-affinity astrocytic Gln transporter (sodium-dependent neutral amino acid transporter, SNAT) (Figure 1). The latter is responsible for the excretion of Gln from astrocytes, the decrease in the expression of which contributes to the retention of Gln in cells and cytotoxic brain edema [21]. Thus, under different scenarios for the development of pathology, several factors can be identified that influence the behavior of keto acids αKG and αKGM. It is possible that the balance of these ketoacids is regulated by those pathophysiological mechanisms that most closely correspond to the development scenario of each specific pathology.

Thus, a number of authors noted that a noticeable increase in the levels of ammonia and Gln was observed in the cerebrospinal fluid (CSF) of patients with comatose liver disease or hyperammonemia [22,23]. At the same time, there was also a significant increase in the level of αKGM, which was almost twice as high as the increase in Gln (~4–5 times versus ≥ 10 times). Therefore, despite the increased levels of Gln and ammonia in the CSF of patients with hyperammonemia, the authors suggested that the level of αKGM correlates better with the level of disease than any other known metabolites [22,23,24,25]. The possibility of αKGM to act as a diagnostic marker in the CSF of patients with hepatic coma, where its levels were many times (~3–10 times) higher than control values, was suggested quite a long time ago [23]. A significant increase in αKGM levels was noted not only in patients with hyperammonemia, but also in children with a urea cycle defect and in patients with a citrine deficiency [26]. Moreover, the mechanism of accumulation of αKGM under these conditions is not yet completely clear. Duffy, and, subsequently, Cooper, suggested that the increase in αKGM levels may be associated either with the inhibition of ω-amidase (ωA) and high levels of Gln in the brain, or with the presence of α-keto acid substrates of glutamine transaminases [24,27]. It is suggested that, to elucidate the mechanism of increased αKGM levels in hyperammonemic diseases, the development of clinically useful tests for the analysis of αKGM, as well as screening for the presence of its potential inhibitors, is necessary [28].

Due to the observed increase in αKGM levels in the CSF of hyperammonemic patients, Vergara et al. first suggested some neurotoxicity of this ketoacid [25]. It was then suggested that the excessive accumulation of αKGM increases the neurotoxicity of ammonia and, thus, contributes to the further development of the pathology [23]. Albrecht and Norenberg believe that Vergara et al.’s findings on the potential role of αKGM in ammonia neurotoxicity are in good agreement with their findings on the role and influence of Gln, which is also involved in this process [29]. The rate of αKGM production is determined by the level of specific activity of glutamine aminotransferase K (GTK), a significant part of which is localized in mitochondria. Mitochondria also contain most of the newly synthesized Gln, which is subsequently metabolized to Glu and ammonia [23,29].

## 4. αKG and αKGM as Potential Biomarkers for the Diagnosis of Liver Diseases

Hyperammonemia is observed in many diseases, including the development of HE. Brusilow and Horwich suggested that hyperammonemia can be conditionally divided into primary and secondary, where primary is more associated with defects in urea cycle enzymes, and secondary is caused directly by the development of HE [30]. At the same time, both diseases have different prerequisites for development, which is often reflected in the metabolic picture of these pathologies. Elevated ammonia levels, for example, are not always associated with the severity of HE. Thus, Ong et al. showed that 60% of patients without symptoms of HE had high ammonia levels, while in patients with advanced HE, ammonia levels were normal or slightly elevated [31]. Similar conclusions were made by Kundra et al. [32]. This may be due to the fact that, in addition to ammonia toxicity, there are other factors that contribute to the development of HE such as, for example, according to Aldridge et al., the presence of inflammatory processes [33].

The first mention of the diagnostic value of the ketoacid αKGM, previously not identified in biological samples, dates back to the 1970s [23,24,25]. This metabolite was found in high concentrations in the CSF of patients with hepatic coma. Vergara et al. showed that, at sufficiently high concentrations (≥10 mM), αKGM can be toxic, which was confirmed by corresponding changes in animal behavior [23]. They suggested that an increased level of αKGM in CSF may be a diagnostic indicator of hepatic coma, and its accumulation may contribute to increased pathogenesis [23]. Subsequently, Duffy et al. showed the presence of αKGM in rat tissues such as the liver, kidney, and brain [24]. The results confirmed the widespread presence of αKGM in biological samples and its association with Gln transamination, as well as explaining the increased levels of αKGM in the CSF of humans with hepatic coma, which was associated with increased use of ammonia for Gln synthesis [24].

Gln transformation can proceed in two, somewhat different, directions. One pathway involves glutaminase-catalyzed hydrolysis to Glu and ammonia, followed by the conversion of Glu to αKG by glutamate dehydrogenase or glutamate-linked aminotransferase (transaminase). The other pathway (glutaminase II pathway) is catalyzed by glutamine transaminase coupled to ωA. The transamination of Gln leads to the formation of αKGM, which, under the influence of ωA, is hydrolyzed to αKG and ammonia [22]. In this chain of reactions, αKGM is one of the important but intermediate metabolites on the path to the formation of αKG. Unfortunately, a rather limited number of publications are devoted to the behavior of αKG in biological samples in liver pathologies. Thus, a decrease in the concentration of αKG below normal in the plasma of children with ornithine transcarbamylase deficiency and patients with urea cycle enzymopathy was previously noted [34]. Moreover, the culmination of the development of acute hyperammonemia in patients occurred only a few days after a decrease in αKG levels. In general, in patients with urea cycle enzymopathies, there was an inverse linear correlation between αKG and ammonia levels (as ammonia levels increased, αKG levels decreased) [34]. It is obvious that excess ammonia, which contributes to a decrease in the level of αKG, in addition to a shift in the balance of the Glu-Gln cycle, can signal a decrease in the functionality of the TCA cycle. It is also interesting that, in patients diagnosed with portosystemic encephalopathy, increasing plasma ammonia levels had no significant effect on changes in αKG levels [34].

Different levels of αKG in patients with liver cirrhosis and portosystemic shunting suggest the presence of different pathophysiological mechanisms in the development of hyperammonemic coma and hepatic coma [34]. Previously, a number of authors drew attention to the increase in the level of αKG in the blood of patients with hepatic coma due to cirrhosis of the liver, and noted that this indicator is highly informative for predicting the outcome of the disease [35,36,37]. Batshaw et al. suggested that the observed difference in the behavior of αKG in patients with hyperammonemic or hepatic coma could be due to different pathophysiological mechanisms of the development of these somewhat similar diseases [34]. The published results mainly concerned studies of the behavior of αKGM or αKG in the CSF, urine, and blood plasma of patients with signs of hyperammonemia, urea cycle enzymopathies, and other severe liver diseases. However, no information was provided on the balance or co-distribution of these metabolites (key participants in the Glu-Gln cycle) in samples from patients or animals with similar but distinct liver pathologies or HE.

A series of publications by Shurubor and co-authors, covering the analysis of a wide range of metabolites, was devoted to filling this gap, as well as to the search for potential markers of the severity of HE development [9,10,11,12]. In samples of the blood plasma, liver, kidney, and brain tissues of rats with varying degrees of HE development, the levels of a number of key metabolites of the Glu-Gln and TCA cycles, including coenzymes and amino and carboxylic acids, as well as the levels of specific activity of the enzymes ωA and GTK, which regulate formation of αKGM and αKG, were discussed. According to the data obtained, the most informative way to assess the severity of HE was the simultaneous measurement of αKG and αKGM levels in rat samples, as well as the assessment of their balance [9,10,11,12]. The balance of keto acids, expressed in the form of the αKG/αKGM ratio, allows one to assess trends in the current state of rats during the development of HE [10,12]. The results of the studies also indicate that the mechanisms of development of hyperammonemia and HE appear to be of a different nature. Thus, if, during hepatic coma and hyperammonemia, the level of the intermediate metabolite of the Glu-Gln cycle, αKGM, increased, and the level of αKG decreased, then, with the development of HE, the trend was exactly the opposite. In samples from rats with chronic HE, αKGM levels decreased and αKG levels increased [12]. In our opinion, this indicates the specific regulation of these ketoacids levels in similar but different liver pathologies.

## 5. Obtaining the αKGM Standard

Unfortunately, to date, the drug αKGM is not commercially available. This significantly complicates experimental work in which there is a need to assess the levels of this ketoacid. Perhaps, the relatively small volume of published works in which the definition of αKGM appears is partly due to this circumstance. There are known methods for the enzymatic or preparative preparation of αKGM, which, however, do not allow us to obtain a sufficient yield of the drug or ensure its required purity.

The method for synthesizing αKGM in vitro was first described by Meister, where αKGM was obtained as a result of enzymatic reactions from L-glutamine [38]. For the oxidative deamination of L-glutamine, the venom of the snake C. adamanteus, which contains L-amino acid oxidase, was used. The synthesis of αKGM was carried out in an aqueous solution in the presence of catalase, which contributed to the removal of hydrogen peroxide formed during the enzymatic reaction. The protein was removed from the resulting solution and the reaction product was purified using a chromatographic column with a cation exchanger in the H^+^ form. The eluate was treated with activated carbon and neutralized with a barium hydroxide solution (barite water). The αKGM solution was concentrated in the barium salt, precipitated with ethanol, and recrystallized. Later, this protocol was slightly modified by Krasnikov et al. [39]. After purification on a cation exchange column, the αKGM solution was decolorized with activated carbon and neutralized with 1 M sodium hydroxide to pH 6.0. The yield of αKGM was 58%; the preparation was slightly contaminated with impurities, in which up to ~5% 5-oxoproline (pyroglutamic acid) and ~1% αKG were found [39].

Relatively recently, Shen et al. proposed a three-stage organic synthesis method for αKGM [40]. The authors used L-2-hydroxyglutamate as the starting material. The steps in the organic synthesis of αKGM began with the introduction of a protecting group on the carboxyl group; then, the hydroxyl group was oxidized using Dess–Martin periodinane and, finally, the protecting group was removed. The product yield was ~53%, and the αKGM preparation was opaque brown crystals. The reliability of the resulting product was confirmed by NMR [40]. Unfortunately, the authors did not provide data on the purity of the resulting product.

A recently published work by Nikulin et al. presented an optimized one-step synthesis of αKGM from L-glutamine catalyzed by *C. adamanteus* L-amino acid oxidase, in which the resulting αKGM had a purity greater than 97% with a final product yield of ≥75% [41]. Moreover, according to this protocol, the αKGM drug can be obtained both in the form of a concentrated aqueous solution and in the form of sodium salt crystals. The method presented by the authors is also applicable for the large-scale synthesis of αKGM (for more details, please see Ref. [41]).

## 6. Methods for the Analysis of αKG and αKGM in Biological Samples

There are no difficulties in determining αKG in biological samples, or in obtaining commercially available standards. To determine αKG in samples, there are various methods based on fluorimetric analysis methods. Liquid chromatography (LC), high-performance liquid chromatography (HPLC), or gas chromatography–mass spectrometry (GC-MS) methods are also known. All these methods differ in that the metabolite requires preliminary derivatization in the presence of O-phenylenediamine [42], phenylhydrazine [43], and O-(2,3,4,5,6-pentafluorobenzyl) hydroxylamine hydrochloride [44]. In relatively simple biological matrices (urine), which usually do not require pretreatment, αKG was determined by liquid chromatography–tandem mass spectrometry (LC-MS/MS) and triple quadrupole MS detection [45].

The determination of αKGM in biological samples has always been associated with certain difficulties due to both the lack of commercially available standards for this keto acid and complex analytical approaches. A review of the available literature revealed only a few available methods for determining αKGM, each of which had its own nuances and/or limitations. These include the measurement of αKGM in human CSF by fluorometric analysis, which requires the use of ωA as an indicator enzyme, the production of which also has its own difficulties [23,25]. In addition, αKGM could not be measured in already deproteinized homogenates, since such extracts have high endogenous fluorescence. Other authors used [14C]KGM and isotope dilution to measure αKGM in rat and human CSF tissues [24]. However, such methods are considered obsolete, and [14C]KGM is currently not commercially available. There is also a fairly sensitive method for determining the αKGM derivative, tris-trimethylsilyl, based on the use of GC/MS, but it also requires serious sample pretreatment and the derivatization of αKGM. This method for determining αKGM was previously used to analyze human urine and CSF [26,46,47].

To simplify the procedure for determining αKG and αKGM in biological samples, Shurubor and co-workers developed a fast and efficient method for the analysis of keto acids, based on HPLC with a UV/VIS detector [48,49]. The analysis protocol includes simple sample preparation, and the method allows for the simultaneous detection of αKG and αKGM in the sample, as well as, if necessary, a number of other Krebs cycle metabolites (for specific details, please see Refs. [48,49]). Combined with the possibility of the laboratory synthesis of pure αKGM standards [41], the presented method for the simultaneous analysis of αKG and αKGM provides additional incentive and opens up new possibilities for the rapid analysis of these keto acids in all types of biological samples, including cells and samples of animal and human fluids and tissues, both in laboratory studies and in clinical trials.

## 7. Distribution Patterns of ωA and GTK in Rat Models of Acute and Chronic HE

As we discussed above, there are two known pathways for the metabolic conversion of Gln to αKG, the major anaplerotic component of the TCA cycle. One of them involves the hydrolysis of Gln to Glu, catalyzed by glutaminases, followed by the conversion of Glu to αKG by Glu-linked aminotransferase or glutamate dehydrogenase. Another pathway for the conversion of Gln to αKG (the glutaminase II pathway) involves the transamination of Gln to αKGM followed by hydrolysis to αKG and ammonia in the presence of ωA. In mammals, this pathway is most well represented in the tissues of the liver and kidneys.

Unfortunately, there are no studies yet that would evaluate the behavior of ωA and GTK regulating the formation of αKG and αKGM in mammals susceptible to HE. Therefore, we are forced to appeal exclusively to the data obtained by Shurubor et al. in rat models of HE caused by the use of TAA [10,11,12]. These studies examined the distribution patterns of the specific activity of the Glu-Gln enzymes of the ωA and GTK cycle, as well as the metabolites αKG and αKGM in samples of the blood plasma and tissues of rats in remission after acute HE and in a state of chronic HE. It is worth taking a closer look at the major differences in the distribution patterns of specific ωA and GTK activity in samples from the rat models considered. Thus, the level of specific activity of these enzymes in samples of the blood plasma and tissues of rats in a state of remission was due to a single dose of TAA in concentrations of 0–200–400–600 mg/kg, and had a slight upward trend in accordance with increasing doses of TAA [11]. Most likely, such a trend in the changes in the specific activity of ωA and GTK was due to processes occurring as a result of the restoration of the functional activity of the liver in rats after acute HE. The exception was brain tissue, where the activity of these enzymes at low and moderate levels of TAA exposure was below the control values and only slightly above them at a high dose of TAA (600 mg/kg). It is interesting that the pattern of changes in the activity of ωA and GTK in brain tissue coincided with the distribution of Gln and the pool of basic amino acids in these rats [11]. It should be noted that, at the lowest doses of TAA (200 mg/kg), the levels of the specific activity of ωA and GTK in the blood plasma, liver, and kidney tissues of rats were close to control values, which may indicate a more rapid recovery after TAA intoxication. In a state of remission after acute HE, the difference in ωA activity in the blood plasma of rats with the highest dose of TAA (600 mg/kg) remained statistically significant [11].

Averaged data on the levels of specific activity of ωA and GTK in groups of rats (200–400–600 mg/kg) in remission after acute HE also indicate its weak growth (~1.1–1.5 times) relative to the control. The exception was brain tissue, where ωA activity in the group of rats with HE remained below control values (~1.2 times), while GTK activity remained almost unchanged (Table 1).

The development of chronic HE over the course of 2–22 weeks otherwise affected the distribution of specific ωA and GTK activities [12]. Thus, the levels of ωA and GTK activity in the liver and kidney tissues of rats were significantly lower (~2–4 times) than control values (Table 1), while, in blood plasma and brain tissues, this decrease was minimal (~1.1 times) [12]. It should be noted that, in the liver of adult rats, the activities of ωA and GTK in the control and TAA-induced groups increased slightly. In the kidney tissues of rats from control groups, the activity of ωA and GTK also slightly increased with age, but decreased with the development of chronic HE [12]. In the brain tissues of aged rats, there was a slight increase in enzyme activity with the development of HE, and in the blood plasma of the same groups of rats there was a slight decrease.

## 8. Distribution Patterns of αKGM and αKG in Rat Models of Acute and Chronic HE

The work of Shurubor et al. showed that, in all samples of rats in remission after the development of acute HE, αKGM levels were reduced relative to the control, regardless of the dose of the toxin received [10]. At the same time, the levels of αKG, in contrast to αKGM, changed in different directions. Thus, in the liver and brain tissues of rats, the levels of αKG increased with increasing TAA dose, and in the blood plasma and kidney tissues of rats, they decreased [10]. The multidirectional trend in the behavior of αKG in rat samples may be due to the stage of remission, during which the recovery of some organs occurred faster than others. Overall, this indicates that level of αKG is different from αKGM in adapting to changes in the pathophysiological state of rats in remission. This may be due to the greater involvement of αKG in the mechanisms of homeostasis regulation, both through participation in the Glu-Gln cycle and the TCA cycle. The increased involvement of αKG in the TCA cycle at the stage of remission in rats after HE may be supported by similar trends in the behavior of metabolites associated with αKG: ACoA, Cit, Iso, Glu, and Gln [9,10,11].

Recent studies have shown that, in experimental groups of rats developing chronic HE, αKGM levels were reduced in all animal plasma and tissue samples compared to control groups of rats [12]. The levels of specific activity of ωA and GTK also decreased (rather than increased, as in post-acute HE). However, αKG levels were increased in all samples from rats with chronic HE. Thus, with a decrease in the levels of specific activity of ωA and GTK in samples of rats with chronic HE by ~1.1–4 times and levels of αKGM by ~1.2–2.5 times, the levels of αKG increased by ~1.5–10 times [12].

Thus, according to previously presented data [10,12], the distribution pattern of αKG and αKGM in rat models of post-acute and chronic HE suggests that the balance of these ketoacids (their ratios) reflects the degree of development of HE and, thus, may act as a potential biomarker in assessing the severity of liver disease. It is proposed to use the ratio of keto acids αKG/αKGM as such an indirect assessment. Table 2 presents data [10,12] on the distribution of αKG and αKGM levels and αKG/αKGM ratios in the blood plasma and tissues of rats with various forms of HE. So, according to Table 2, the αKG/αKGM ratio in the blood plasma of rats with chronic HE was increased relative to control values by ~15 times, in liver tissue by ~14 times, in kidney tissue by ~4.5 times, and in brain tissue by ~2 times. It should be noted, here, that the calculated values of αKG/αKGM for the blood plasma and liver of rats with chronic HE are close. From the point of view of monitoring the condition of rats with a chronic form of HE, it can be assumed that the close values of these ratios in the blood plasma and liver of rats allow us to limit ourselves to collecting blood plasma samples instead of liver tissue and, thus, provide the opportunity to extend work with the same group of animals.

Similar calculations performed for animals at different stages of post-acute HE (Table 2) indicate values of αKG/αKGM ratios in brain tissue comparable with chronic HE. Perhaps this indicates the presence of irreversible or not completely reversible pathological changes in the brain of rats even after a period of rehabilitation. In addition, according to the relative values of the αKG/αKGM ratio in the groups of TAA-induced and control rats, the complete restoration of the metabolic balance was not observed in the liver and kidneys of the animals. At the same time, a noticeable restoration of metabolic balance was noted in the blood plasma of rats in a state of remission. The comparison of αKG/αKGM ratios in rat samples from post-acute and chronic models of HE indicates that the restoration of the functional activity of rat organs after TAA intoxication occurred at different rates. In the blood plasma, positive changes occurred much faster than in the tissues of the kidneys, liver, and brain of rats. Thus, by changing the balance of αKG/αKGM in the blood plasma of rats in remission, one can monitor the improvement of their condition. At the same time, the change in the balance of these keto acids in the internal organs of rats at the remission stage was not so noticeable. According to Díez-Fernández et al., the complete recovery of the liver of rats after TAA intoxication occurs after 72 h (3 days) [50]. However, in the works of Shurubor et al., the duration of the experiment with the remission of rats after acute TAA-induced HE was 6 days and, according to the authors’ conclusion, even this time is not enough to completely restore the metabolic balance of animals [9,10,11,12].

## 9. The Need for Additional Experiments to Clarify the Mechanisms of the Development of Liver Pathologies

The analysis of the distribution of αKG and αKGM in samples of blood plasma and animal tissues with various liver pathologies revealed two differently directed trends, which are obviously associated with the nuances of the disease or the underlying causes that caused it [10,12,22,23,24,25,26,27,28,34,35,36,37]. Thus, in reported cases of hyperammonemia due to enzymopathy or similar pathologies, Kuhara et al. noted an increase in αKGM levels in patient’s urine samples [26]. Other authors have also noted a decrease in patient’s plasma αKG levels with the development of hyperammonemia [34,35,36,37]. Thus, in contrast to the increase in αKGM levels in the CSF and urine of patients with hyperammonemia or comatose liver disease, in rats with various forms of HE, a consistent decrease in αKGM levels was observed in all analyzed samples (blood plasma, liver, kidney, and brain) [10,12,22].

Data on the nature of the simultaneous distribution of keto acids αKG and αKGM, linked by a single Glu-Gln cycle, both in hyperammonemia and in the case of TAA-induced HE, were not found in open sources before the publications of Shurubor and co-authors [10,12].

As noted earlier, the reason for the identified behavior of αKG and αKGM in HE and hyperammonemia may be existing differences in the underlying causes and pathophysiology of the diseases, which affect changes in the activity of the corresponding enzymes and the work of the Glu-Gln cycle and the TCA cycle. However, there is a small possibility that this may also be due to differences in the nature of the samples analyzed (human urine/CSF vs blood plasma, liver, kidney, and rat brain tissue).

To understand the mechanisms of pathophysiology affecting the behavior of αKG and αKGM, as well as Gln and ammonia in fluids and tissues of humans or animals with liver pathologies, it is desirable to conduct unified studies that, if possible, would rely on both the analysis of the same types of samples and a list of specific metabolites important for monitoring the progression of the disease. Thus, if, in the study of hyperammonemic conditions, the subject of study was usually the CSF and urine of patients and, less often, blood plasma, then, in other studies, during the development of TAA-induced HE, metabolic changes in the blood plasma and tissues of rats were considered. Therefore, drawing any parallels between the studies conducted is possible only with certain reservations.

When comparing the results of the analysis of CSF and blood plasma, it is necessary to take into account the existing difference between these fluids. CSF is a plasma ultrafiltrate contained in the ventricles of the brain and subarachnoid spaces of the skull and spine, which ensures the transport of nutrients to the neurons of the brain and the excretion of metabolic products of nervous tissues [51]. Since the composition of CSF is strictly regulated, any changes in it can be useful for diagnostic purposes. According to published data, the levels of metabolites in CSF and circulating blood plasma do not always coincide and can sometimes differ, which is obviously due to the specificity of their compartments [52]. In particular, such differences have been noted in patients with progressive neurodegenerative diseases (Huntington’s disease and Parkinson’s disease) [53,54]. We suggest that, possibly, the most likely reason for the discovery of opposite trends in the distribution of αKGM in hyperammonemia and HE is not the type of samples analyzed, but the mechanisms of the development of these pathologies, including the functioning of the Glu-Gln cycle. However, this assumption requires further research.

In general, hyperammonemia and HE can be considered as two borderline conditions that, under certain circumstances, can develop independently or synchronously [30,31,33]. With hyperammonemia, a metabolic disorder occurs due to a lack of urea cycle enzymes, which is accompanied by the subsequent poisoning of the body with ammonia. Liver damage inducing the development of HE is associated with a number of neuropsychiatric manifestations, where hyperammonemia may be one of the drivers of the disease. There is evidence that, in some patients with HE, the level of ammonia in the blood does not increase, and other triggers of the disease take part in the pathogenesis: cytokines immune factors of the blood, brain, etc. Therefore, the role of ammonia in the disease HE is still controversial [55].

In this regard, I would like to note that, in the works of Shurubor and co-authors on the search for specific biomarkers of the disease in rat models with post-acute and chronic HE, the emphasis was shifted towards expanding the range of analyzed metabolites in the blood plasma and tissues of animals [9,10,11,12]. As a result, the potential diagnostic value of the simultaneous determination of two keto acids, αKG and αKGM, as well as their αKG/αKGM ratio, in animal samples with HE was noted. The obtained result differs from previously published data, which is most likely due to the different metabolic background of pathologies such as HE and hyperammonemia.

## 10. Conclusions

The presented work examines some aspects of the involvement of the Glu-Gln cycle keto acids, αKG and αKGM, in the development of HE and evaluates the results of experimental and clinical studies, as well as the prerequisites for the use of these keto acids as diagnostic markers of the disease. It has been suggested that, in rat models of TAA-induced HE, ketoacids αKG and αKGM are more associated with the degree of disease development than other metabolites.

The distribution of metabolites αKG and αKGM in the blood plasma and organ tissues of rats is determined both by the severity of the course of HE and the degree of their recovery at the stage of remission after acute HE. It is assumed that the αKG/αKGM ratio in the blood plasma of rats with chronic HE reflects the degree of liver disease and can act as a potential biomarker for diagnosing the severity of this pathology. We believe that, in the obtained approach to monitoring the degree of HE development in TAA-induced rat models, it makes sense to test on the clinical plasma samples of patients with a confirmed diagnosis of HE disease. If successful, this indicator could be recommended for consideration for use as a biomarker both for monitoring the recovery process of patients after acute HE and for assessing the severity of their chronic disease.

## Figures and Tables

**Table 1:** Levels of specific activity of ωA and GTK (mean value * ± standard deviation, nmol/mg/min) in blood plasma, liver, kidney, and brain tissues of control and TAA-induced rats.

	Blood Plasma		Liver		Kidneys		Brain	
	Control	HE	Control	HE	Control	HE	Control	HE
Remission period after acute HE								
ωA	0.0322 ± 0.0153	0.0473 ± 0.0091	0.0711 ± 0.0172	0.0729 ± 0.0210	0.0459 ± 0.0158	0.0525 ± 0.0121	0.0060 ± 0.0022	0.0049 ± 0.0026
GTK	N/D	N/D	0.0050 ± 0.0005	0.0056 ± 0.0010	0.0084 ± 0.0029	0.0086 ± 0.0020	0.0023 ± 0.0002	0.0023 ± 0.0006
Chronic HE								
ωA	0.0154 ± 0.0035	0.0145 ± 0.0066	0.0672 ± 0.0048	0.0241 ± 0.0060 **	0.0729 ± 0.0126	0.0433 ± 0.0123 **	0.0045 ± 0.0015	0.0044 ± 0.0017
GTK	0.0029 ± 0.0014	0.0032 ± 0.0023	0.0044 ± 0.0002	0.0013 ± 0.0002 **	0.0118 ± 0.0015	0.0046 ± 0.0013 **	0.0020 ± 0.0002	0.0018 ± 0.0002

* The average values include all groups of rats (data from sources [11,12]). ** Significantly different compared to control; *p* < 0.05.

**Table 2:** Levels of αKG and αKGM (mean * ± standard deviation) in plasma, liver, kidney, and brain tissues of control and TAA-induced rats.

	Blood Plasma ^1^		Liver ^2,3^		Kidneys ^2,3^		Brain ^2,3^	
	Control	HE	Control	HE	Control	HE	Control	HE
Remission period after acute HE								
αKG	1.84 ± 0.38	0.96 ± 0.45	0.08 ± 0.02	0.41 ± 0.19 **	0.14 ± 0.18	0.08 ± 0.05	0.05 ± 0.07	0.10 ± 0.04
αKGM	18.88 ± 1.20	15.67 ± 3.49	1.64 ± 0.39	0.81 ± 0.29 **	0.11 ± 0.04	0.03 ± 0.03 **	0.04 ± 0.01	0.03 ± 0.02
αKG/αKGM	0.10	0.06	0.05	0.51	1.27	2.66	1.25	3.33
R	0.6		10.2		2.1		2.6	
Chronic HE								
αKG	2.80 ± 2.56	19.07 ± 7.09 **	0.001 ± 0.000	0.01 ± 0.00 **	0.22 ± 0.36	0.40 ± 0.45	0.005 ± 0.006	0.007 ± 0.006
αKGM	21.2 ± 6.37	9.70 ± 4.51 **	0.80 ± 0.20	0.71 ± 0.16	0.29 ± 0.07	0.12 ± 0.04 **	0.30 ± 0.22	0.23 ± 0.05
αKG/αKGM	0.13	1.97	0.001	0.014	0.76	3.33	0.016	0.030
R	15.2		14.0		4.4		1.9	

^1^—blood plasma, μM. ^2^—tissues of the liver, kidneys, and brain, nmol/mg protein (acute HE). ^3^—liver, kidney, and brain tissue, nmol/mg raw tissue (chronic HE). R—multiplicity of αKG/αKGM levels in tissue samples of rats with HE in relation to the control. * data for all groups of TAA-induced rats (acute and chronic HE) are summarized into one group (data taken from sources [10,12]). ** Significantly different compared to control; *p* < 0.05.

## Data Availability

Data can be made available upon personal request.
